# Bornological stabilization families and oscillation dynamics of closed-set sequences

**DOI:** 10.1371/journal.pone.0359280

**Published:** 2026-09-28

**Authors:** Zhi-Peng Lin, Mehmet Gürdal, Ömer Kişi

**Affiliations:** 1 School of Artificial Intelligence, Xiamen University Tan Kah Kee College, Xiamen, Fujian, China; 2 Department of Mathematics, Süleyman Demirel University, Isparta, Turkey; 3 Department of Mathematics, Bartin University, Bartın, Turkey; Northeast Normal University, CHINA

## Abstract

Convergence of moving closed sets is usually studied relative to a family of test regions fixed in advance. This may conceal the regions on which a given sequence actually stabilizes. We introduce a sequence-dependent construction in a metric space. For each test set, we take the infimum over all tails of the supremum of the symmetric localized excess between pairs of tail members. The test sets for which this quantity is zero form the stabilization family. We prove that this family is hereditary and closed under finite unions, and hence is a bornology whenever it covers the ambient space. Under neighborhood stabilization and a compactness condition preventing local traces from escaping, we obtain either eventual disappearance from a test region or convergence to a nonempty local closed limit. A nested countable cofinal family then yields compatible local limits and a global cluster set. When this set is nonempty, bornological convergence follows. We further prove that every preassigned bornology supporting neighborhood convergence is contained in the generated neighborhood-stabilization family. Counterexamples separate the construction from global Hausdorff convergence and pointwise convergence of distance functions, and establish that compactness is indispensable. The novelty is that the testing bornology is derived from the tail behavior of the sequence itself. This construction may be useful for moving feasible sets and set-valued approximations that stabilize locally but not globally.

## 1 Introduction

The convergence of closed subsets of metric spaces is commonly studied through a mode of convergence chosen in advance. Hausdorff-type and Painlevé–Kuratowski-type convergence are classical tools in hyperspace theory and variational analysis; see, for example [1,2]. Wijsman convergence is determined by the pointwise convergence of the distance functions associated with moving closed sets [3]. Variational, graphical, and epigraphical convergence provide related methods for functions, operators, and set-valued mappings [2,4].

Bornological convergence replaces global comparison by comparison on a prescribed family of test sets. In this setting, a bornology is fixed first, and the convergence of the sequence is then examined relative to that testing structure. The resulting lower and upper convergences, together with their topological properties, have been studied using excesses, distance functions, and metric enlargements [5–7]. A recent monograph provides a systematic treatment of bornologies in metric analysis [8], while recent work has further clarified the relations among several forms of set convergence induced by an arbitrary bornology [9]. These theories are particularly useful when the behavior of unbounded sets must be examined on bounded or otherwise designated regions of the ambient space.

A separate line of research concerns localized sequences of points. There, one determines the subsets on which scalar distance sequences satisfy convergence or Cauchy conditions. Such questions have been studied in metric spaces and related settings [10,11]. Ideal versions of localized convergence were considered in [12]. These notions are based primarily on the scalar sequences obtained by evaluating distances at fixed points.

The problem considered here reverses the usual order of construction. Let $\left(A_{n})\subseteq CL(X\right)$ be a sequence of nonempty closed subsets of a metric space (*X*,*d*). Instead of prescribing a bornology before examining the sequence, we determine the subsets S⊆X on which the pairwise localized excesses of $A_{m}$ and $A_{n}$ tend to zero as m,n→∞. Thus, the admissible testing family is derived from the asymptotic behavior of the sequence itself. This sequence-dependent choice is the principal distinction between the present study and the fixed-bornology formulations in the existing literature.

For each test set *S*, we introduce a symmetric localized excess and the corresponding tail oscillation index. The sets on which this index vanishes form the stabilization family. We prove that this family is hereditary and stable under finite unions; consequently, it is a bornology whenever it covers the ambient space. Vanishing tail oscillation alone, however, does not ensure the existence of a closed limit. We therefore combine neighborhood stabilization with strong neighborhood trace-compactness, a condition that prevents the relevant traces from escaping every compact subset of the space.

Under these assumptions, we first recover a local cluster set on each closed testing region. The local result gives a precise alternative: either the traces are eventually empty, or they converge to a nonempty closed local limit with respect to the localized excess. We then prove that the recovered limits agree on nested test sets. An increasing countable cofinal family of closed regions allows these compatible local limits to be identified with a global cluster set. When the global cluster set is nonempty, the sequence converges bornologically with respect to the stabilization bornology generated by the sequence.

We also compare the generated family with a bornology chosen independently of the sequence. The maximality theorem shows that every prescribed bornology with respect to which the sequence is neighborhood-bornologically convergent is contained in the neighborhood-stabilization family. Thus, the generated family contains every testing bornology that already supports convergence of the sequence. The examples show that this conclusion is not a reformulation of global Hausdorff convergence or pointwise convergence of distance functions. They also demonstrate that the compactness assumption in the recovery theorem cannot be omitted.

The results may be useful when moving feasible sets, approximation sets, or solution sets exhibit stable behavior on selected regions but fail to converge globally. Such situations arise naturally in variational analysis, set-valued optimization, and approximation procedures involving unbounded sets. The stabilization family identifies the regions on which the sequence is asymptotically Cauchy, while the recovery theorems specify the additional compactness needed to obtain a closed limit. These observations provide a direct interpretation of the abstract results without asserting applications that are not established in the present paper.

The paper is organized as follows. Section 2 introduces the localized excess, the tail oscillation index, and the stabilization families. Section 3 establishes the local and global recovery theorems, the compatibility result, and the maximality theorem. Section 4 presents examples that compare the proposed definitions with Hausdorff and Wijsman-type convergence and clarify the role of the compactness assumption. The final section summarizes the conclusions and indicates directions for further study.

## 2 Oscillation gauges and stabilization families

This section introduces the quantities used throughout the paper. We first define a localized excess for two closed sets on a prescribed test region. Its symmetric form is then used to define a tail oscillation index for sequences of closed sets. The sets on which this index vanishes constitute the stabilization family.

Throughout the paper, (*X*,*d*) denotes a metric space,


$$\begin{array}{c} CL(X)=\{A\subseteq X:\;A\text{≠}\emptyset \text{ and }A\text{ is closed}\}, \end{array}$$

and


$$\begin{array}{c} {CL}_{0}(X)=\{A\subseteq X:\;A\text{ is closed}\}. \end{array}$$

For x∈X and E⊆X, we set


$$\begin{array}{c} d(x,E):=\inf_{y\in E}d(x,y), \end{array}$$

with the convention d(x,∅)=+∞. For E⊆X and ε>0, we write


$$\begin{array}{c} E^{\varepsilon }:=\{x\in X:\;d(x,E)<\varepsilon \} \end{array}$$

for the open ε-enlargement of *E*.

Our first task is to define a localized gauge that measures how far two closed sets are from one another on a prescribed test region.

**Definition 2.1.** Let A,C∈CL(X) and let S⊆X. The localized excess of *A* over *C* on *S* is defined by


$$\begin{array}{c} e_{S}(A,C):=\sup_{a\in A\cap S}d(a,C), \end{array}$$

with the convention that $e_{S}(A,C)=0$ whenever A∩S=∅.

*Remark* 2.1. The quantity $e_{S}(A,C)$ measures the distance from the points of *A* lying in *S* to the set *C*. It is generally asymmetric. We set $e_{S}(A,C)=0$ when A∩S=∅, since in that case *A* has no points to be tested on *S*.

**Definition 2.2.** Let A,C∈CL(X) and let S⊆X. We define


$$\begin{array}{c} \Delta_{S}(A,C):=\max\{e_{S}(A,C),\;e_{S}(C,A)\}. \end{array}$$

We call $\Delta_{S}(A,C)$ the localized oscillation gauge of *A* and *C* on *S*.

The quantity $\Delta_{S}(A,C)$ plays the role of a local symmetric excess distance. It does not compare the sets globally, but only through their behavior inside the test region *S*.

*Remark* 2.2. The gauges $e_{S}(A,C)$ and $\Delta_{S}(A,C)$ are defined in this paper for nonempty closed sets, that is, for A,C∈CL(X). In the recovery theory, however, the local or global cluster set produced by the sequence may be empty. In that case, the correct interpretation is not a literal $\Delta_{S}$-convergence to the empty set, but the eventual disappearance of the relevant traces from the testing region. Accordingly, empty recovered limits will be treated separately in Section 3.

We now pass from two sets to a sequence of sets. The following tail quantity is the main numerical invariant of the theory.

**Definition 2.3.** Let $\left(A_{n})\subseteq CL(X\right)$ and let S⊆X. The bornological oscillation index of $\left(A_{n}\right)$ on *S* is


$$\begin{array}{c} \omega_{S}(A_{n}):=\underset{N\in \mathbb{N}}{\inf}\;\underset{m,n\geq N}{\sup}\Delta_{S}(A_{m},A_{n}). \end{array}$$

Equivalently,


$$\begin{array}{c} \omega_{S}(A_{n})=\lim_{N\to \infty }\underset{m,n\geq N}{\sup}\Delta_{S}(A_{m},A_{n}), \end{array}$$

since the tail suprema form a decreasing sequence in *N*.

*Remark* 2.3. The quantity $\omega_{S}(A_{n})$ is a localized tail Cauchy index. It is defined without specifying a candidate limit. The equality $\omega_{S}(A_{n})=0$ means that $\Delta_{S}(A_{m},A_{n})$ tends to zero as m,n→∞.

This leads to the central object of the paper.

**Definition 2.4.** Let $\left(A_{n})\subseteq CL(X\right)$. The bornological stabilization family generated by $\left(A_{n}\right)$ is


$$\begin{array}{c} S_{stab}(A_{n}):=\{S\subseteq X:\;\omega_{S}(A_{n})=0\}. \end{array}$$

If $S_{stab}(A_{n})$ covers *X*, that is,


$$\begin{array}{c} X=\underset{S\in S_{stab}(A_{n})}{⋃}S, \end{array}$$

then we call $S_{stab}(A_{n})$ the stabilization bornology of $\left(A_{n}\right)$.

Local recovery requires control not only on the test set itself but also on its arbitrarily small metric enlargements. This leads to the following strengthened form of stabilization.

**Definition 2.5.** Let $\left(A_{n})\subseteq CL(X\right)$. A set S⊆X is called a neighborhood-stabilizing region for $\left(A_{n}\right)$ if $\omega_{S^{\eta }}(A_{n})=0$ for every η>0. The family of all neighborhood-stabilizing regions is denoted by


$$\begin{array}{c} S_{nstab}(A_{n}):=\left\{S\subseteq X:\;\omega_{S^{\eta }}(A_{n})=0\text{ for every }\eta >0\right\}. \end{array}$$

Thus $S_{nstab}(A_{n})$ records stabilization that is stable under all metric neighborhood enlargements of the testing region.

**Proposition 2.1.** *Let* $\left(A_{n})\subseteq CL(X\right)$. *Then* $S_{nstab}(A_{n})$ *is hereditary and stable under finite unions. Consequently, if* $S_{nstab}(A_{n})$ *covers X, then it is a bornology on X.*

*Proof.* Let T⊆S and assume that $S\in S_{nstab}(A_{n})$. For every η>0, we have $T^{\eta }\subseteq S^{\eta }.$ By the monotonicity of the oscillation index with respect to the testing set,


$$\begin{array}{c} \omega_{T^{\eta }}(A_{n})\leq \omega_{S^{\eta }}(A_{n})=0. \end{array}$$

Hence $T\in S_{nstab}(A_{n})$.

Now let $S,T\in S_{nstab}(A_{n})$. Since


$$\begin{array}{c} (S\cup T{)}^{\eta }=S^{\eta }\cup T^{\eta }\;(\eta >0), \end{array}$$

and since Proposition 2.2 gives finite-union stability of the stabilization family, we obtain


$$\begin{array}{c} \omega_{(S\cup T{)}^{\eta }}(A_{n})=\omega_{S^{\eta }\cup T^{\eta }}(A_{n})=\max\left\{\omega_{S^{\eta }}(A_{n}),\omega_{T^{\eta }}(A_{n})\right\}=0. \end{array}$$

Therefore $S\cup T\in S_{nstab}(A_{n})$.

The final assertion follows from the hereditary property, finite-union stability, and the covering property. □

The terminology is deliberate. The family $S_{stab}(A_{n})$ is not fixed in advance; it is created by the asymptotic behavior of the sequence. The next proposition shows that this family already has the correct hereditary and finite-union structure expected of a bornological object.

**Proposition 2.2.** *Let* $\left(A_{n})\subseteq CL(X\right)$*. Then the stabilization family* $S_{stab}(A_{n})$ *has the following properties:*

(i) *If* T⊆S *and* $S\in S_{stab}(A_{n})$*, then* $T\in S_{stab}(A_{n})$*.*
(ii) *If* $S,T\in S_{stab}(A_{n})$*, then* $S\cup T\in S_{stab}(A_{n})$*.*

*In particular,* $S_{stab}(A_{n})$ *is a hereditary family stable under finite unions.*

*Proof.* For (i), let T⊆S. Then, for every A,C∈CL(X),


$$\begin{array}{c} e_{T}(A,C)\leq e_{S}(A,C),\;e_{T}(C,A)\leq e_{S}(C,A), \end{array}$$

and hence $\Delta_{T}(A,C)\leq \Delta_{S}(A,C).$ Therefore,


$$\begin{array}{c} \underset{m,n\geq N}{\sup}\Delta_{T}(A_{m},A_{n})\leq \underset{m,n\geq N}{\sup}\Delta_{S}(A_{m},A_{n}) \end{array}$$

for every N∈ℕ. Taking infima over *N* yields $\omega_{T}(A_{n})\leq \omega_{S}(A_{n}).$ Thus $\omega_{S}(A_{n})=0$ implies $\omega_{T}(A_{n})=0$, so $T\in S_{stab}(A_{n})$.

For (ii), observe first that for all A,C∈CL(X),


$$\begin{array}{c} e_{S\cup T}(A,C)=\underset{a\in A\cap (S\cup T)}{\sup}d(a,C)=\max\{e_{S}(A,C),\;e_{T}(A,C)\}. \end{array}$$

By symmetry,


$$\begin{array}{c} e_{S\cup T}(C,A)=\max\{e_{S}(C,A),\;e_{T}(C,A)\}, \end{array}$$

and hence


$$\begin{array}{c} \Delta_{S\cup T}(A,C)=\max\{\Delta_{S}(A,C),\;\Delta_{T}(A,C)\}. \end{array}$$

It follows that for every N∈ℕ,


$$\begin{array}{c} \sup_{m,n\geq N}\Delta_{S\cup T}(A_{m},A_{n})=\max\left\{\sup_{m,n\geq N}\Delta_{S}(A_{m},A_{n}),\sup_{m,n\geq N}\Delta_{T}(A_{m},A_{n})\right\}. \end{array}$$

Now both tail sequences are decreasing in *N*, so passing to infima gives


$$\begin{array}{c} \omega_{S\cup T}(A_{n})=\max\{\omega_{S}(A_{n}),\;\omega_{T}(A_{n})\}. \end{array}$$

Therefore, if $\omega_{S}(A_{n})=\omega_{T}(A_{n})=0$, then $\omega_{S\cup T}(A_{n})=0$. Thus $S\cup T\in S_{stab}(A_{n})$. □

**Corollary 2.1.** *If* $S_{stab}(A_{n})$ *covers X, then it is a bornology on X.*

*Proof.* By Proposition 2.2, the family is hereditary and stable under finite unions. If it covers *X*, then it satisfies the defining cover property of a bornology. □

Proposition 2.2 shows that the stabilization family is an ideal of subsets of *X*. If it covers *X*, it is a bornology. To obtain closed limits from this family, an additional compactness assumption is required.

**Definition 2.6.** Let $\left(A_{n})\subseteq CL(X\right)$ and let F⊆P(X). We say that $\left(A_{n}\right)$ is strongly neighborhood trace-compact on F if for every S∈F there exists a compact set $K_{S}\subseteq X$ such that for every ε>0 one has


$$\begin{array}{c} A_{n}\cap S^{\varepsilon }\subseteq K_{S}^{\varepsilon } \end{array}$$

for all sufficiently large *n*.

*Remark* 2.4. Strong neighborhood trace-compactness prevents the points of $A_{n}$ lying near *S* from leaving every compact subset of *X*. The same compact set $K_{S}$ is used for every neighborhood radius. This uniformity allows convergent subsequences to be selected in the proofs below.

## 3 Recovery of bornological limits from stabilization

The stabilization family identifies the regions on which the sequence is asymptotically Cauchy with respect to the localized gauge. This condition does not by itself provide a closed limit. In this section, we combine neighborhood stabilization with strong neighborhood trace-compactness to obtain local and global limits.

We first prove a local result on a closed test set. The compactness assumption provides convergent subsequences, while neighborhood stabilization ensures that the resulting cluster set is independent of the selected subsequences.

We begin with four auxiliary lemmas. Lemma 3.1 gives a localized excess estimate on an enlarged test set. This enlargement is necessary because a point selected from an intermediate set need not belong to the original test region. The remaining lemmas provide neighborhood control and the compactness properties used in the recovery theorems.

**Lemma 3.1.** *Let* S⊆X *and let* A,B,C∈CL(X)*. If* $e_{S}(A,B)<r<\infty ,$ *then* $e_{S}(A,C)\leq r+e_{S^{r}}(B,C).$ *Consequently, if* $\max\{e_{S}(A,B),e_{S}(C,B)\}<r<\infty ,$ *then*


$$\begin{array}{c} \Delta_{S}(A,C)\leq r+\max\left\{e_{S^{r}}(B,C),e_{S^{r}}(B,A)\right\}. \end{array}$$

*Proof.* If A∩S=∅, then $e_{S}(A,C)=0$, and the first assertion is immediate. Assume that A∩S≠∅ and fix x∈A∩S. Since $r>e_{S}(A,B)$, we have


$$\begin{array}{c} d(x,B)\leq e_{S}(A,B)<r. \end{array}$$

Hence there exists y∈B such that *d*(*x*,*y*)<*r*. Because x∈S, this implies $y\in S^{r}.$ Therefore


$$\begin{array}{c} d(x,C)\leq d(x,y)+d(y,C)<r+e_{S^{r}}(B,C). \end{array}$$

Taking the supremum over all x∈A∩S, we obtain


$$\begin{array}{c} e_{S}(A,C)\leq r+e_{S^{r}}(B,C). \end{array}$$

Now assume that $r>\max\{e_{S}(A,B),e_{S}(C,B)\}.$ Applying the first part to the triple (*A*,*B*,*C*) gives


$$\begin{array}{c} e_{S}(A,C)\leq r+e_{S^{r}}(B,C). \end{array}$$

Applying the first part to the triple (*C*,*B*,*A*) gives


$$\begin{array}{c} e_{S}(C,A)\leq r+e_{S^{r}}(B,A). \end{array}$$

Taking the maximum of these two estimates yields


$$\begin{array}{c} \Delta_{S}(A,C)\leq r+\max\left\{e_{S^{r}}(B,C),e_{S^{r}}(B,A)\right\}. \end{array}$$

This completes the proof. □

To connect convergence with the tail condition defining stabilization, we first record the following implication.

**Lemma 3.2.** *Let* T⊆X*, let* $\left(A_{n})\subseteq CL(X\right)$*, and let* A∈CL(X)*. Assume that* $\Delta_{T^{\eta }}(A_{n},A)⟶0$ *for every* η>0. *Then* $\omega_{T}(A_{n})=0.$

*Proof.* Let ε>0 and put r=ε/3. By assumption, there exists N∈ℕ such that


$$\begin{array}{c} \Delta_{T^{r}}(A_{k},A)<r \end{array}$$

for every k≥N. Since $T\subseteq T^{r}$, it follows that


$$\begin{array}{c} \max\{e_{T}(A_{m},A),e_{T}(A_{n},A)\}<r \end{array}$$

whenever m,n≥N. Lemma 3.1, applied to the triple $\left(A_{m},A,A_{n}\right)$, now gives


$$\begin{array}{c} \Delta_{T}(A_{m},A_{n})\leq r+\max\{e_{T^{r}}(A,A_{n}),e_{T^{r}}(A,A_{m})\}<2r<\varepsilon . \end{array}$$

Hence $\omega_{T}(A_{n})=0$. □

The compactness assumption enters the recovery argument through the following subsequence criterion.

**Lemma 3.3.** *Let* S⊆X *be closed and assume that* $\left(A_{n}\right)$ *is strongly neighborhood trace-compact on {S}. If* $\left(x_{n}\right)$ *is a sequence such that* $x_{n}\in A_{n}\cap S^{\eta_{n}}$ *for all sufficiently large n, where* $\eta_{n}↓0$*, then* $\left(x_{n}\right)$ *admits a convergent subsequence whose limit belongs to S.*

*Proof.* Let $K_{S}$ be the compact set given by strong neighborhood trace-compactness. Choose indices $n_{1}<n_{2}<\cdots$ so large that


$$\begin{array}{c} \eta_{n_{k}}<\frac{1}{k}\;\text{and}\;A_{n_{k}}\cap S^{1/k}\subseteq K_{S}^{1/k} \end{array}$$

for every *k*. Since $x_{n_{k}}\in A_{n_{k}}\cap S^{\eta_{n_{k}}}\subseteq A_{n_{k}}\cap S^{1/k}$, we have $x_{n_{k}}\in K_{S}^{1/k}.$ Choose $y_{k}\in K_{S}$ such that $d(x_{n_{k}},y_{k})<\frac{1}{k}.$ By compactness of $K_{S}$, the sequence $\left(y_{k}\right)$ has a convergent subsequence, say $y_{k_{j}}\to y\in K_{S}$. Then


$$\begin{array}{c} d(x_{n_{k_{j}}},y)\leq d(x_{n_{k_{j}}},y_{k_{j}})+d(y_{k_{j}},y)\to 0, \end{array}$$

so $x_{n_{k_{j}}}\to y$. Since $x_{n_{k_{j}}}\in S^{\eta_{n_{k_{j}}}}$ and $\eta_{n_{k_{j}}}\to 0$, we obtain *d*(*y*,*S*)=0. Because *S* is closed, y∈S. □

We next extract the uniform tail estimate supplied by neighborhood stabilization.

**Lemma 3.4.** *Let* S⊆X *and let* $\left(A_{n})\subseteq CL(X\right)$*. Assume that* $\omega_{S^{\eta }}(A_{n})=0$ *for every* η>0. *Then for every* ε>0 *there exists* $N_{\varepsilon }\in \mathbb{N}$ *such that* $\Delta_{S^{\varepsilon }}(A_{m},A_{n})<\varepsilon$ *whenever* $m,n\geq N_{\varepsilon }.$ *In particular,*


$$\begin{array}{c} A_{m}\cap S^{\varepsilon }\subseteq A_{n}^{\varepsilon }\;\text{and}\;A_{n}\cap S^{\varepsilon }\subseteq A_{m}^{\varepsilon } \end{array}$$

*for all* $m,n\geq N_{\varepsilon }.$

*Proof.* Fix ε>0. Since $\omega_{S^{\varepsilon }}(A_{n})=0$, there exists $N_{\varepsilon }\in \mathbb{N}$ such that $\Delta_{S^{\varepsilon }}(A_{m},A_{n})<\varepsilon$ for all $m,n\geq N_{\varepsilon }.$ By the definition of $\Delta_{S^{\varepsilon }}$, this means


$$\begin{array}{c} e_{S^{\varepsilon }}(A_{m},A_{n})<\varepsilon \;\text{and}\;e_{S^{\varepsilon }}(A_{n},A_{m})<\varepsilon \end{array}$$

for all $m,n\geq N_{\varepsilon }$. Hence every point of $A_{m}\cap S^{\varepsilon }$ lies within distance ε of $A_{n}$, and vice versa. □

The preceding estimates now allow the local cluster set to be recovered on a closed neighborhood-stabilizing region.

**Theorem 3.1.** *Let (X,d) be a metric space and let* $\left(A_{n})\subseteq CL(X\right)$*. Let* S⊆X *be a closed set such that* $\omega_{S^{\eta }}(A_{n})=0$ *for every* η>0. *Assume that* $\left(A_{n}\right)$ *is strongly neighborhood trace-compact on {S}. Define*


$$\begin{array}{c} A_{S}:=\{x\in S:\;\exists \;n_{k}\to \infty ,\;\exists \;x_{k}\in A_{n_{k}}\text{ such that }x_{k}\to x\}. \end{array}$$

*Then* $A_{S}\in {CL}_{0}(X)$, $A_{S}\subseteq S$*, and exactly one of the following alternatives holds:*

(i) $A_{S}=\emptyset$*, in which case* $A_{n}\cap S=\emptyset$ *for all sufficiently large n;*
(ii) $A_{S}\text{≠}\emptyset$*, in which case* $A_{S}\in CL(X)$ *and* $\Delta_{S}(A_{n},A_{S})⟶0$ *as* n→∞.

*Moreover, the recovered set is unique in the following neighborhood sense: if* B∈CL(X), B⊆S*, and* $\Delta_{S^{\eta }}(A_{n},B)⟶0$ *for every* η>0, *then* $B=A_{S}$.

*Proof.* By definition, $A_{S}\subseteq S$. We first show that $A_{S}$ is closed. Let $(z_{j})\subseteq A_{S}$ and suppose that $z_{j}\to z$ in *X*. Since *S* is closed, we have z∈S. For each *j*, choose an index $n_{j}\geq j$ and a point $u_{j}\in A_{n_{j}}$ such that $d(u_{j},z_{j})<\frac{1}{j}.$ Then


$$\begin{array}{c} d(u_{j},z)\leq d(u_{j},z_{j})+d(z_{j},z)\to 0. \end{array}$$

Thus $u_{j}\to z$, and hence $z\in A_{S}$. Therefore $A_{S}\in {CL}_{0}(X)$. Strong neighborhood trace-compactness is inherited by every subsequence of $\left(A_{n}\right)$, a fact that will be used below.

Assume first that $A_{S}=\emptyset$. If $A_{n}\cap S\text{≠}\emptyset$ for infinitely many *n*, choose a subsequence $\left(n_{k}\right)$ and points $x_{k}\in A_{n_{k}}\cap S$. Apply Lemma 3.3 to the subsequence $\left(A_{n_{k}}\right)$, the points $\left(x_{k}\right)$, and the radii $\eta_{k}=1/k$. Indeed, $x_{k}\in S\subseteq S^{1/k}$ for every *k*. The sequence $\left(x_{k}\right)$ therefore has a convergent subsequence whose limit belongs to *S*. This limit belongs to $A_{S}$, a contradiction. Hence $A_{n}\cap S=\emptyset$ for all sufficiently large *n*.

Assume now that $A_{S}\text{≠}\emptyset$. Then $A_{S}\in CL(X)$. We first prove that


$$\begin{array}{c} e_{S}(A_{n},A_{S})\to 0. \end{array}$$

Suppose, to the contrary, that there exist $\varepsilon_{0}>0$, a subsequence $\left(n_{k}\right)$, and points $x_{k}\in A_{n_{k}}\cap S$ such that $d(x_{k},A_{S})\geq \varepsilon_{0}$ for all *k*. Applying Lemma 3.3 to the subsequence $\left(A_{n_{k}}\right)$, the points $\left(x_{k}\right)$, and the radii $\eta_{k}=1/k$, we may pass to a further subsequence and assume that $x_{k}\to x$ for some x∈S. Since $x_{k}\in A_{n_{k}}$ and $n_{k}\to \infty$, the definition of $A_{S}$ gives $x\in A_{S}$. This contradicts


$$\begin{array}{c} d(x_{k},A_{S})\leq d(x_{k},x)\to 0. \end{array}$$

Thus $e_{S}(A_{n},A_{S})\to 0$.

Next we prove that $e_{S}(A_{S},A_{n})\to 0.$ Suppose not. Then there exist $\varepsilon_{0}>0$, a subsequence $\left(n_{k}\right)$, and points $x_{k}\in A_{S}\subseteq S$ such that $d(x_{k},A_{n_{k}})\geq \varepsilon_{0}$ for all *k*. Put $\delta =\varepsilon_{0}/3$. Since $\omega_{S^{\delta }}(A_{n})=0$, there exists $N_{\delta }\in \mathbb{N}$ such that


$$\begin{array}{c} \Delta_{S^{\delta }}(A_{m},A_{n})<\delta \end{array}$$

whenever $m,n\geq N_{\delta }.$ After discarding finitely many terms, we may assume that $n_{k}\geq N_{\delta }$ for every *k*. For each *k*, since $x_{k}\in A_{S}$, there exist arbitrarily large indices *m* and points $y\in A_{m}$ such that $d(y,x_{k})<\delta .$ Choose $m_{k}\geq \max\{n_{k},k,N_{\delta }\}$ and $y_{k}\in A_{m_{k}}$ such that $d(y_{k},x_{k})<\delta .$ Since $x_{k}\in S$, we have $y_{k}\in S^{\delta }$. Therefore $y_{k}\in A_{m_{k}}\cap S^{\delta }.$ By the choice of $N_{\delta }$,


$$\begin{array}{c} d(y_{k},A_{n_{k}})<\delta . \end{array}$$

Consequently,


$$\begin{array}{c} d(x_{k},A_{n_{k}})\leq d(x_{k},y_{k})+d(y_{k},A_{n_{k}})<2\delta <\varepsilon_{0}, \end{array}$$

which contradicts the choice of $x_{k}$. Thus $e_{S}(A_{S},A_{n})\to 0$.

Combining the two estimates gives $\Delta_{S}(A_{n},A_{S})\to 0.$

It remains to prove the stated neighborhood uniqueness. Let B∈CL(X), B⊆S, and assume that $\Delta_{S^{\eta }}(A_{n},B)\to 0$ for every η>0. First let b∈B. Since B⊆S, we have $b\in S^{\eta }$ for every η>0. Taking η=1/k, choose $n_{k}\geq k$ so large that


$$\begin{array}{c} e_{S^{1/k}}(B,A_{n_{k}})<\frac{1}{k}. \end{array}$$

Then there exists $b_{k}\in A_{n_{k}}$ such that $d(b,b_{k})<\frac{1}{k}.$ Hence $b_{k}\to b$, and therefore $b\in A_{S}$. Thus $B\subseteq A_{S}$.

Conversely, let $x\in A_{S}$. Then there exist $n_{k}\to \infty$ and $x_{k}\in A_{n_{k}}$ such that $x_{k}\to x$. Fix η>0. Since x∈S, for all sufficiently large *k* one has $x_{k}\in S^{\eta }$. Since


$$\begin{array}{c} e_{S^{\eta }}(A_{n_{k}},B)\to 0, \end{array}$$

we obtain $d(x_{k},B)\to 0.$ Because *B* is closed and $x_{k}\to x$, it follows that x∈B. Hence $A_{S}\subseteq B$. Therefore $B=A_{S}$. □

The second step is global. Once local limits exist on sufficiently many stabilizing regions, one may ask whether they are compatible and can be assembled into a global cluster set on the whole space. For this purpose, we use an increasing countable cofinal family of closed test sets. Countability coordinates the local comparisons, while nesting makes the recovered sets compatible.

**Theorem 3.2.** *Let (X,d) be a metric space and let* $\left(A_{n})\subseteq CL(X\right)$*. Let* $S_{i}\subseteq S_{j}$ *be closed sets such that* $\omega_{S_{i}^{\eta }}(A_{n})=0$ *and* $\omega_{S_{j}^{\eta }}(A_{n})=0$ *for every* η>0. *Assume that* $\left(A_{n}\right)$ *is strongly neighborhood trace-compact on* $\left\{S_{i},S_{j}\right\}$*. Let* $A_{S_{i}}$ *and* $A_{S_{j}}$ *be the local limits given by Theorem 3.1. Then* $A_{S_{i}}=A_{S_{j}}\cap S_{i}.$

*Proof.* Let $x\in A_{S_{i}}$. Then $x\in S_{i}\subseteq S_{j}$ and, by definition of $A_{S_{i}}$, there exist integers $n_{k}\to \infty$ and points $x_{k}\in A_{n_{k}}$ such that $x_{k}\to x.$ Since $x\in S_{j}$, the same subsequential cluster relation shows that $x\in A_{S_{j}}$. Hence $A_{S_{i}}\subseteq A_{S_{j}}\cap S_{i}.$

Conversely, let $x\in A_{S_{j}}\cap S_{i}$. Since $x\in A_{S_{j}}$, there exist integers $n_{k}\to \infty$ and points $x_{k}\in A_{n_{k}}$ such that $x_{k}\to x.$ Because $x\in S_{i}$, this is exactly the defining condition for *x* to belong to $A_{S_{i}}$. Therefore


$$\begin{array}{c} A_{S_{j}}\cap S_{i}\subseteq A_{S_{i}}. \end{array}$$

The proof is complete. □

The compatibility relation permits the local cluster sets to be identified with the restrictions of a single global cluster set. An increasing countable cofinal family supplies one ordered index for the diagonal construction, while cofinality transfers the resulting convergence to every stabilizing test set.

**Theorem 3.3.** *Let (X,d) be a metric space and let* $\left(A_{n})\subseteq CL(X\right)$*. Assume that there exists an increasing sequence of closed sets* $S_{1}\subseteq S_{2}\subseteq \cdots$ *such that*


$$\begin{array}{c} X=\overset{\infty }{\underset{j=1}{⋃}}S_{j}, \end{array}$$

*and such that the following conditions hold:*

(i) $\omega_{S_{j}^{\eta }}(A_{n})=0$ *for every* j∈ℕ *and every* η>0;
(ii) $\left(A_{n}\right)$ *is strongly neighborhood trace-compact on* $\left\{S_{j}:j\in \mathbb{N}\right\}$;
(iii) *for every* $S\in S_{stab}(A_{n})$ *there exists* j∈ℕ *such that* $S\subseteq S_{j}.$

*Define*


$$\begin{array}{c} A:=\{x\in X:\;\exists \;n_{k}\to \infty ,\;\exists \;x_{k}\in A_{n_{k}}\text{ such that }x_{k}\to x\}. \end{array}$$

*Then* $A\in {CL}_{0}(X)$ *and, for every* j∈ℕ, $A\cap S_{j}=A_{S_{j}},$ *where* $A_{S_{j}}$ *is the local cluster set obtained in Theorem 3.1. Moreover, exactly one of the following holds:*

1. A=∅*, in which case* $A_{n}\cap S=\emptyset$ *for every* $S\in S_{stab}(A_{n})$ *and all sufficiently large n;*
2. A∈CL(X)*, in which case* $\Delta_{S}(A_{n},A)\to 0$ *for every* $S\in S_{stab}(A_{n})$*. Moreover,* $\Delta_{S^{\eta }}(A_{n},A)\to 0$ *for every* $S\in S_{nstab}(A_{n})$ *and every* η>0. *Hence* $\left(A_{n}\right)$ *converges to A with respect to the neighborhood-stabilization bornology in the neighborhood-bornological sense. In this sense, A is the unique nonempty closed set in* CL(X) *with the above neighborhood-bornological convergence property.*

*Proof.* We first show that *A* is closed. Let $(z_{j})\subseteq A$ and suppose that $z_{j}\to z$ in *X*. For each *j*, choose an index $n_{j}\geq j$ and a point $u_{j}\in A_{n_{j}}$ such that $d(u_{j},z_{j})<\frac{1}{j}.$ Then


$$\begin{array}{c} d(u_{j},z)\leq d(u_{j},z_{j})+d(z_{j},z)\to 0, \end{array}$$

so $u_{j}\to z$. Hence z∈A, and therefore $A\in {CL}_{0}(X)$. Condition (i) implies that $S_{j}\in S_{nstab}(A_{n})$ for every j∈ℕ. Since the sets $S_{j}$ cover *X*, Proposition 2.1 shows that $S_{nstab}(A_{n})$ is a bornology. Moreover, $S_{nstab}(A_{n})\subseteq S_{stab}(A_{n})$, so the stabilization family also covers *X* and is a bornology.

Fix j∈ℕ. We prove that $A\cap S_{j}=A_{S_{j}}.$ If $x\in A\cap S_{j}$, then by definition of *A* there exist integers $n_{k}\to \infty$ and points $x_{k}\in A_{n_{k}}$ such that $x_{k}\to x.$ Since $x\in S_{j}$, the defining property of $A_{S_{j}}$ is satisfied, so $x\in A_{S_{j}}$. Thus $A\cap S_{j}\subseteq A_{S_{j}}.$

Conversely, if $x\in A_{S_{j}}$, then by definition of $A_{S_{j}}$ there exist integers $n_{k}\to \infty$ and points $x_{k}\in A_{n_{k}}$ such that $x_{k}\to x.$ Hence x∈A, and since $x\in S_{j}$, we obtain $x\in A\cap S_{j}.$ Therefore $A_{S_{j}}\subseteq A\cap S_{j},$ and hence $A\cap S_{j}=A_{S_{j}}.$

Assume first that A=∅. Then $A_{S_{j}}=A\cap S_{j}=\emptyset$ for every j∈ℕ. By Theorem 3.1(i), $A_{n}\cap S_{j}=\emptyset$ for all sufficiently large *n*. Now let $S\in S_{stab}(A_{n})$. By assumption, there exists j∈ℕ such that $S\subseteq S_{j}.$ Hence


$$\begin{array}{c} A_{n}\cap S\subseteq A_{n}\cap S_{j}=\emptyset \end{array}$$

for all sufficiently large *n*, which proves (i).

We now assume that A≠∅, so A∈CL(X). Fix j∈ℕ. If $A\cap S_{j}=\emptyset ,$ then $A_{S_{j}}=\emptyset ,$ and Theorem 3.1(i) gives $A_{n}\cap S_{j}=\emptyset$ for all sufficiently large *n*. Since also $A\cap S_{j}=\emptyset$, it follows that


$$\begin{array}{c} e_{S_{j}}(A_{n},A)=0\;\text{and}\;e_{S_{j}}(A,A_{n})=0 \end{array}$$

for all sufficiently large *n*, hence $\Delta_{S_{j}}(A_{n},A)\to 0.$

If instead $A\cap S_{j}\text{≠}\emptyset ,$ then


$$\begin{array}{c} A_{S_{j}}=A\cap S_{j}\in CL(X), \end{array}$$

and Theorem 3.1(ii) yields $\Delta_{S_{j}}(A_{n},A_{S_{j}})\to 0.$ Since $A_{S_{j}}=A\cap S_{j}$, we have


$$\begin{array}{c} e_{S_{j}}(A,A_{n})=e_{S_{j}}(A_{S_{j}},A_{n}), \end{array}$$

and because $A_{S_{j}}\subseteq A$,


$$\begin{array}{c} e_{S_{j}}(A_{n},A)\leq e_{S_{j}}(A_{n},A_{S_{j}}). \end{array}$$

Therefore $\Delta_{S_{j}}(A_{n},A)\to 0.$

Thus, in all cases, $\Delta_{S_{j}}(A_{n},A)\to 0$ for every j∈ℕ.

Now let $S\in S_{stab}(A_{n})$. By assumption, there exists j∈ℕ such that $S\subseteq S_{j}.$ By monotonicity of the localized gauge,


$$\begin{array}{c} \Delta_{S}(A_{n},A)\leq \Delta_{S_{j}}(A_{n},A)\to 0. \end{array}$$

Hence $\Delta_{S}(A_{n},A)\to 0$ for every $S\in S_{stab}(A_{n})$, so $\left(A_{n}\right)$ bornologically converges to *A* with respect to its stabilization bornology.

Now let $S\in S_{nstab}(A_{n})$ and let η>0. By definition of $S_{nstab}(A_{n})$, we have $\omega_{S^{\eta }}(A_{n})=0.$ Thus $S^{\eta }\in S_{stab}(A_{n})$. By the cofinality assumption, there exists j∈ℕ such that $S^{\eta }\subseteq S_{j}.$ Using the already proved convergence on $S_{j}$, we obtain


$$\begin{array}{c} \Delta_{S^{\eta }}(A_{n},A)\leq \Delta_{S_{j}}(A_{n},A)\to 0. \end{array}$$

Therefore $\Delta_{S^{\eta }}(A_{n},A)\to 0$ for every $S\in S_{nstab}(A_{n})$ and every η>0. It remains to prove uniqueness in the neighborhood-bornological sense. Let B∈CL(X) be another nonempty closed set such that $\Delta_{S^{\eta }}(A_{n},B)\to 0$ for every $S\in S_{nstab}(A_{n})$ and every η>0.

First let x∈A. Choose j∈ℕ such that $x\in S_{j}$. By the definition of *A*, there exist integers $n_{k}\to \infty$ and points $x_{k}\in A_{n_{k}}$ such that $x_{k}\to x$. Fix η>0. Since $x\in S_{j}$, we have $x_{k}\in S_{j}^{\eta }$ for all sufficiently large *k*. Since $\Delta_{S_{j}^{\eta }}(A_{n},B)\to 0,$ it follows that $d(x_{k},B)\to 0.$ Because *B* is closed and $x_{k}\to x$, we obtain x∈B. Hence A⊆B.

Conversely, let b∈B. Choose j∈ℕ such that $b\in S_{j}$. For each k∈ℕ, choose $n_{k}\geq k$ so large that


$$\begin{array}{c} e_{S_{j}^{1/k}}(B,A_{n_{k}})<\frac{1}{k}. \end{array}$$

Since $b\in B\cap S_{j}^{1/k}$, there exists $y_{k}\in A_{n_{k}}$ such that $d(b,y_{k})<\frac{1}{k}.$ Thus $y_{k}\to b$ and $n_{k}\to \infty$. By the definition of the global cluster set *A*, we obtain b∈A. Hence B⊆A. Therefore *A* = *B*. □

Theorem 3.3 combines the local limits associated with a countable cofinal family and identifies them with the global cluster set of the sequence. If this set is nonempty, the theorem also gives bornological convergence with respect to the generated stabilization bornology.

The compactness assumption cannot be omitted. Vanishing oscillation does not exclude the possibility that the relevant points of the sequence leave every compact subset of the space. Strong neighborhood trace-compactness rules out this behavior.

It remains to compare the induced stabilization bornology with externally prescribed bornologies in the classical sense. The next result shows that every such bornology along which the sequence already converges must be contained in the stabilization family.

**Definition 3.1.** Let B be a bornology on *X*, let $\left(A_{n})\subseteq CL(X\right)$, and let A∈CL(X). We say that $\left(A_{n}\right)$ is neighborhood-bornologically convergent to *A* with respect to B if $\Delta_{S^{\eta }}(A_{n},A)⟶0$ for every S∈B and every η>0. In this case we write $A_{n}\overset{nb\;}{\underset{B\;}{\to }}A.$

The preceding definition allows the generated testing family to be compared with every bornology prescribed independently of the sequence.

**Theorem 3.4.** *Let* B *be a bornology on X and let* $\left(A_{n})\subseteq CL(X\right)$*. Assume that there exists* A∈CL(X) *such that* $A_{n}\overset{nb\;}{\underset{B\;}{\to }}A.$ *Then* $B\subseteq S_{nstab}(A_{n}).$ *Equivalently, every test set on which the sequence is neighborhood-bornologically convergent belongs automatically to the neighborhood-stabilization family generated by the sequence.*

*Proof.* Let S∈B. We prove that $S\in S_{nstab}(A_{n})$. Fix η>0. We need to show that


$$\begin{array}{c} \omega_{S^{\eta }}(A_{n})=0. \end{array}$$

Let ρ>0 be arbitrary. Since $(S^{\eta }{)}^{\rho }\subseteq S^{\eta +\rho },$ we have


$$\begin{array}{c} \Delta_{(S^{\eta }{)}^{\rho }}(A_{n},A)\leq \Delta_{S^{\eta +\rho }}(A_{n},A). \end{array}$$

By the neighborhood-bornological convergence assumption, $\Delta_{S^{\eta +\rho }}(A_{n},A)\to 0.$ Hence $\Delta_{(S^{\eta }{)}^{\rho }}(A_{n},A)\to 0$ for every ρ>0. Applying Lemma 3.2 with $T=S^{\eta }$, we obtain $\omega_{S^{\eta }}(A_{n})=0.$ Since η>0 was arbitrary, $S\in S_{nstab}(A_{n})$. Therefore

$B\subseteq S_{nstab}(A_{n}).$ □

**Corollary 3.1.** *Assume that the hypotheses of Theorem 3.3 hold and that the recovered global cluster set satisfies* A∈CL(X)*. Then the neighborhood-stabilization bornology* $S_{nstab}(A_{n})$ *is maximal among all bornologies* B *on X for which there exists a nonempty closed set* B∈CL(X) *such that* $A_{n}\overset{nb\;}{\underset{B\;}{\to }}B.$ *In particular, for every such bornology* B *one has* $B\subseteq S_{nstab}(A_{n}).$

*Proof.* This follows directly from Theorem 3.4. □

**Corollary 3.2.** *Assume that the sequence* $\left(A_{n})\subseteq CL(X\right)$ *satisfies the recovery hypotheses of Theorem 3.3 with respect to two increasing closed cofinal bases*


$$\begin{array}{c} S_{1}\subseteq S_{2}\subseteq \cdots ,\;X=\overset{\infty }{\underset{j=1}{⋃}}S_{j}, \end{array}$$

*and*


$$\begin{array}{c} T_{1}\subseteq T_{2}\subseteq \cdots ,\;X=\overset{\infty }{\underset{k=1}{⋃}}T_{k}, \end{array}$$

*both subordinate to the stabilization family. Let A and B be the corresponding global cluster sets obtained in Theorem 3.3. Then A = B. Hence the recovered global cluster set is independent of the chosen countable cofinal base.*

*Proof.* By Theorem 3.3, both *A* and *B* coincide with the set of all subsequential cluster points of $\left(A_{n}\right)$ in *X*. Since this set is determined solely by the sequence and not by the chosen cofinal exhaustion, we conclude that *A* = *B*. □

*Remark* 3.1. The countable cofinal base is used to pass from compatible local limits to a limit on the whole space. The global cluster set is defined directly in terms of subsequential limits of points from $\left(A_{n}\right)$ and therefore does not depend on the particular cofinal base used in the proof.

**Corollary 3.3.** *Let (X,d) be a proper metric space and let* $\left(A_{n})\subseteq CL(X\right)$*. Assume that there exists an increasing sequence of closed bounded sets*


$$\begin{array}{c} S_{1}\subseteq S_{2}\subseteq \cdots ,\;X=\overset{\infty }{\underset{j=1}{⋃}}S_{j}, \end{array}$$

*such that* $\omega_{S_{j}^{\eta }}(A_{n})=0$ *for every* j∈ℕ *and every* η>0. *Assume moreover that for every* j∈ℕ *there exists a bounded set* $B_{j}\subseteq X$ *such that for every* ε>0 *there exists* $N_{j,\varepsilon }\in \mathbb{N}$ *with* $A_{n}\cap S_{j}^{\varepsilon }\subseteq B_{j}^{\varepsilon }$ *for all* $n\geq N_{j,\varepsilon }.$ *Then* $\left(A_{n}\right)$ *is strongly neighborhood trace-compact on* $\left\{S_{j}:j\in \mathbb{N}\right\}$.

*Consequently, the hypotheses of Theorem 3.3 are satisfied whenever every* $S\in S_{stab}(A_{n})$ *is contained in some* $S_{j}$*. In that case, the sequence has a closed global cluster set* $A\in {CL}_{0}(X)$ *consisting of all subsequential limits of points selected from the sets* $A_{n}$*. If* A≠∅*, then* $\Delta_{S}(A_{n},A)\to 0$ *for every* $S\in S_{stab}(A_{n}),$ *and hence* $\left(A_{n}\right)$ *bornologically converges to A with respect to its stabilization bornology.*

*Proof.* Fix j∈ℕ. Since *X* is proper and $B_{j}$ is bounded, the closure $K_{j}:=\overset{―}{B_{j}^{\;1}}$ is compact. Let ε>0 be given. If 0<ε≤1, then for all sufficiently large *n*,


$$\begin{array}{c} A_{n}\cap S_{j}^{\varepsilon }\subseteq B_{j}^{\varepsilon }\subseteq K_{j}^{\varepsilon }. \end{array}$$

If ε>1, then again


$$\begin{array}{c} A_{n}\cap S_{j}^{\varepsilon }\subseteq B_{j}^{\varepsilon }\subseteq K_{j}^{\varepsilon } \end{array}$$

for all sufficiently large *n*. Hence for every ε>0, $A_{n}\cap S_{j}^{\varepsilon }\subseteq K_{j}^{\varepsilon }$ for all sufficiently large *n*. Therefore $\left(A_{n}\right)$ is strongly neighborhood trace-compact on $\left\{S_{j}:j\in \mathbb{N}\right\}$.

The remaining assertions follow directly from Theorem 3.3. □

## 4 Examples and counterexamples

This section gives examples illustrating the preceding definitions and results. The first example shows stabilization on every bounded test set without global Hausdorff convergence. The second shows that neighborhood stabilization does not imply recovery when strong neighborhood trace-compactness fails. The third illustrates the compatibility of local limits on nested test sets. The final example separates stabilization from pointwise convergence of distance functions.

**Example 4.1.** Let X=ℝ with the usual metric, and consider the sequence of closed sets


$$\begin{array}{c} A_{n}:=[n,\infty )\in CL(\mathbb{R}),\;n\in \mathbb{N}. \end{array}$$

We show that $\left(A_{n}\right)$ admits a nontrivial stabilization structure on bounded regions, while it fails to converge in the global Hausdorff sense.

First observe that every bounded set belongs to the neighborhood-stabilization family. Let S⊆ℝ be bounded and fix η>0. Choose *M* > 0 such that


$$\begin{array}{c} S^{\eta }\subseteq [-M,M]. \end{array}$$

If *n* > *M*, then $A_{n}\cap S^{\eta }=\emptyset$. Hence, for all sufficiently large *m*,*n*,


$$\begin{array}{c} A_{m}\cap S^{\eta }=\emptyset \;\text{and}\;A_{n}\cap S^{\eta }=\emptyset , \end{array}$$

and therefore


$$\begin{array}{c} \Delta_{S^{\eta }}(A_{m},A_{n})=0. \end{array}$$

Thus $\omega_{S^{\eta }}(A_{n})=0$ for every η>0. Consequently, every bounded set belongs to $S_{nstab}(A_{n})$ and hence also to $S_{stab}(A_{n})$.

The increasing family


$$\begin{array}{c} S_{j}:=[-j,j],\;j\in \mathbb{N}, \end{array}$$

is a closed exhaustion of ℝ consisting of neighborhood-stabilizing bounded sets, and


$$\begin{array}{c} \mathbb{R}=\overset{\infty }{\underset{j=1}{⋃}}S_{j}. \end{array}$$

This family is cofinal among the bounded subsets of ℝ, although it need not be cofinal in the full stabilization family $S_{stab}(A_{n})$. Moreover, $\left(A_{n}\right)$ is strongly neighborhood trace-compact on $\left\{S_{j}:j\in \mathbb{N}\right\}$. Indeed, if ε>0 is fixed and n>j+ε, then


$$\begin{array}{c} A_{n}\cap S_{j}^{\varepsilon }=\emptyset , \end{array}$$

so the required inclusion holds for any compact set $K_{S_{j}}$.

We now determine the local and global cluster sets. If x∈ℝ, no sequence $x_{k}\in A_{n_{k}}$ with $n_{k}\to \infty$ can converge to *x*, since $x_{k}\geq n_{k}\to \infty$. Hence the global cluster set is empty. The local cluster set associated with each $S_{j}$ is also empty. Thus the sequence stabilizes on every bounded test region, although it has no nonempty global cluster set.

On the other hand, $\left(A_{n}\right)$ is not Hausdorff convergent in CL(ℝ). Indeed, for *m* > *n* one has $h(A_{n},A_{m})=m-n,$ where *h* denotes the Hausdorff distance between closed subsets of ℝ. In particular, $\left(A_{n}\right)$ is not Cauchy in this metric, and therefore it cannot converge in the Hausdorff sense.

This example shows that stabilization on bounded test sets does not imply global Hausdorff convergence. The sets eventually leave every bounded region, whereas their pairwise Hausdorff distances remain unbounded.

The next example shows why the compactness assumption in the recovery theorem cannot be removed.

**Example 4.2.** Let X=ℚ be equipped with the metric inherited from ℝ, and choose a sequence of rational numbers $\left(q_{n}\right)$ such that $q_{n}\to \sqrt{2}$ in ℝ. For each n∈ℕ, define


$$\begin{array}{c} A_{n}:=\{q_{n}\}\in CL(\mathbb{Q}). \end{array}$$

We show that the sequence is neighborhood-stabilizing on the whole space but does not satisfy strong neighborhood trace-compactness. Consequently, the hypotheses of Theorem 3.1 are not satisfied, and no nonempty closed limit exists in ℚ.

Since X=ℚ, we have $X^{\eta }=X$ for every η>0. Moreover, for all m,n∈ℕ,


$$\begin{array}{c} \Delta_{X}(A_{m},A_{n})=|q_{m}-q_{n}|. \end{array}$$

Because $\left(q_{n}\right)$ is a Cauchy sequence in ℚ, it follows that $\omega_{X}(A_{n})=0.$ Hence $X\in S_{stab}(A_{n}).$ In particular, the sequence exhibits zero oscillation on a large stabilizing region, namely the whole ambient space.

We claim, however, that $\left(A_{n}\right)$ is not strongly neighborhood trace-compact on {*X*}. Assume to the contrary that there exists a compact set K⊆ℚ such that for every ε>0 one has


$$\begin{array}{c} A_{n}\cap X^{\varepsilon }=A_{n}\subseteq K^{\varepsilon }\;\text{for all sufficiently large }n. \end{array}$$

Since $A_{n}=\{q_{n}\}$, this means precisely that $d(q_{n},K)\to 0.$ Now *K* is compact in ℚ, hence also compact in ℝ. Passing to the limit along $q_{n}\to \sqrt{2}$ in ℝ, we obtain $d(\sqrt{2},K)=0.$ Because *K* is compact in ℝ, it is closed in ℝ, and therefore $\sqrt{2}\in K.$ This is impossible since K⊆ℚ. Thus no such compact set *K* exists, and strong neighborhood trace-compactness fails.

We next show that there is no nonempty closed set A∈CL(ℚ) such that $\Delta_{X}(A_{n},A)\to 0.$ Assume such a set *A* exists. Since


$$\begin{array}{c} e_{X}(A,A_{n})=\sup_{a\in A}|a-q_{n}|\to 0, \end{array}$$

we claim that *A* must be a singleton. Indeed, if a,b∈A, then


$$\begin{array}{c} |a-b|\leq |a-q_{n}|+|q_{n}-b|\leq 2e_{X}(A,A_{n})\to 0, \end{array}$$

so *a* = *b*. Hence *A*={*a*} for some a∈ℚ. But then


$$\begin{array}{c} \Delta_{X}(A_{n},A)=|q_{n}-a|\to 0, \end{array}$$

so $q_{n}\to a$ in ℚ, which contradicts $q_{n}\to \sqrt{2}$ in ℝ.

Therefore $\left(A_{n}\right)$ is neighborhood-stabilizing on *X*, but strong neighborhood trace-compactness fails and there is no nonempty closed set A∈CL(ℚ) such that $\Delta_{X}(A_{n},A)\to 0$. This proves that vanishing oscillation alone is not sufficient for recovery.

We now illustrate how the local limits agree when the test regions are nested.

**Example 4.3.** Let X=ℝ with the usual metric, and define


$$\begin{array}{c} A_{n}:=\left\{\frac{1}{n},\;1+\frac{1}{n},\;n\right\}\in CL(\mathbb{R}),\;n\in \mathbb{N}. \end{array}$$

Consider the two closed sets


$$\begin{array}{c} S_{1}:=\left[-\frac{1}{2},\frac{1}{2}\right],\;S_{2}:=\left[-\frac{1}{2},\frac{3}{2}\right]. \end{array}$$

Then $S_{1}\subseteq S_{2}.$

We claim that $\left(A_{n}\right)$ is neighborhood-stabilizing on both *S*_1_ and *S*_2_, and that the corresponding local limits satisfy the compatibility relation $A_{S_{1}}=A_{S_{2}}\cap S_{1}.$

We first identify the local limits. Since $\frac{1}{n}\to 0,$ $1+\frac{1}{n}\to 1,$ n→∞, the only subsequential cluster points of $\left(A_{n}\right)$ lying in *S*_1_ are given by 0. Hence $A_{S_{1}}=\{0\}.$ On the other hand, the subsequential cluster points of $\left(A_{n}\right)$ lying in *S*_2_ are precisely 0 and 1. Therefore


$$\begin{array}{c} A_{S_{2}}=\{0,1\}. \end{array}$$

It follows immediately that


$$\begin{array}{c} A_{S_{2}}\cap S_{1}=\{0\}=A_{S_{1}}, \end{array}$$

which is exactly the conclusion of Theorem 3.2.

We verify the stabilization assumptions. Fix η>0. Since $S_{1}^{\eta }$ and $S_{2}^{\eta }$ are bounded, the point *n* eventually lies outside both sets. The point 1/*n* eventually belongs to both neighborhoods. For $S_{1}^{\eta }$, the point 1 + 1/*n* is eventually excluded when η≤1/2 and eventually included when η>1/2. For $S_{2}^{\eta }$, both 1/*n* and 1 + 1/*n* eventually belong to the testing region. In each case, every point that remains in the relevant trace belongs to a convergent branch. It follows that


$$\begin{array}{c} \omega_{S_{1}^{\eta }}(A_{n})=0\;\text{and}\;\omega_{S_{2}^{\eta }}(A_{n})=0 \end{array}$$

for every η>0.

Moreover, the sequence is strongly neighborhood trace-compact on $\left\{S_{1},S_{2}\right\}$. Indeed, for each i∈{1,2} one may choose a compact interval $K_{i}$ containing $S_{i}^{1}\cup \{0,1\}$, and then for every ε>0 all sufficiently large traces $A_{n}\cap S_{i}^{\varepsilon }$ are contained in $K_{i}^{\varepsilon }$.

Thus the smaller test set gives the local limit {0}, whereas the larger test set gives {0,1}. Their restrictions agree on the smaller region, as stated in Theorem 3.2.

*Remark* 4.1. Example 4.3 shows that enlarging the test set may add new cluster points. Theorem 3.2 ensures that the local limits agree on the smaller test set.

The final example separates trace stabilization from pointwise convergence of distance functions.

**Example 4.4.** Let X=ℝ with the usual metric, and consider the sequence of closed sets


$$\begin{array}{c} A_{n}:=\left\{1+\frac{(-1{)}^{n}}{2}\right\}\in CL(\mathbb{R}),\;n\in \mathbb{N}. \end{array}$$

Thus $A_{2n}=\left\{\frac{3}{2}\right\}$ and $A_{2n+1}=\left\{\frac{1}{2}\right\}.$

Let $S:=\left[-\frac{1}{4},\frac{1}{4}\right].$ Since $A_{n}\cap S=\emptyset$ for every *n*, we have


$$\begin{array}{c} e_{S}(A_{m},A_{n})=0\;\text{and}\;e_{S}(A_{n},A_{m})=0 \end{array}$$

for all m,n∈ℕ. Hence $\Delta_{S}(A_{m},A_{n})=0$ for all m,n∈ℕ, and therefore $\omega_{S}(A_{n})=0.$ It follows that $S\in S_{stab}(A_{n}).$

On the other hand, the pointwise distance sequence at x=0∈S is


$$\begin{array}{c} d(0,A_{2n})=\frac{3}{2},\;d(0,A_{2n+1})=\frac{1}{2}. \end{array}$$

Thus the scalar sequence $d(0,A_{n})$ does not converge. In fact, for every $x\in \left[-\frac{1}{4},\frac{1}{4}\right]$ one has


$$\begin{array}{c} d(x,A_{2n})=\left|x-\frac{3}{2}\right|,\;d(x,A_{2n+1})=\left|x-\frac{1}{2}\right|, \end{array}$$

so the pointwise distance profile oscillates on the whole region *S* and has no limit there.

Therefore the stabilization behavior on *S* and the pointwise distance-profile behavior on *S* lead to completely different conclusions:


$$\begin{array}{c} \omega_{S}(A_{n})=0,\;\text{whereas}\;d(x,A_{n})\text{ fails to converge for every }x\in S. \end{array}$$

This example shows that stabilization on a test set does not imply pointwise convergence of the distance functions on that set. The localized gauge uses only the traces $A_{n}\cap S$, whereas Wijsman convergence examines the values $d(x,A_{n})$ for fixed points *x*.

*Remark* 4.2. The example also shows the effect of the empty-trace convention. Although $A_{n}\cap S=\emptyset$ for every n, the distances from points of S to $A_{n}$ may still oscillate.

## 5 Conclusion and further directions

We introduced a sequence-dependent procedure for identifying the test regions on which a sequence of nonempty closed sets has vanishing tail oscillation. The resulting stabilization family is hereditary and stable under finite unions, and it becomes a bornology whenever it covers the ambient space. The original feature of the construction is that this bornology is derived from the sequence rather than prescribed beforehand.

Neighborhood stabilization alone does not ensure the existence of a closed limit. Under strong neighborhood trace-compactness, however, each closed test region has either an eventually empty trace or a nonempty recovered local limit. The local limits agree on nested regions. A countable cofinal family then determines a global cluster set and yields bornological convergence whenever that set is nonempty. The maximality theorem further shows that every preassigned bornology supporting neighborhood-bornological convergence is contained in the generated neighborhood-stabilization family.

The examples establish the limits of these conclusions. Local stabilization may hold without global Hausdorff convergence, and it does not imply pointwise convergence of distance functions. Thus, the sequence-generated method is not a reformulation of either fixed-bornology convergence or pointwise distance-function convergence. The examples also show that the compactness assumption is indispensable: vanishing oscillation cannot prevent local traces from escaping every compact subset of the space.

For moving feasible sets, approximation sets, and solution sets in variational problems, the construction can identify regions of stable behavior even when no global convergence is available. Further work may examine weaker compactness conditions, relations with Kuratowski, Wijsman, and Attouch–Wets convergence, and quantitative estimates based on the tail oscillation index.
