# Supplementary material for: Bornological stabilization families and oscillation dynamics of closed-set sequences
Source: PLoS One. 2026 Sep 28;21(9):e0359280. doi: 10.1371/journal.pone.0359280 (PMC13618950; doi:10.1371/journal.pone.0359280)
Supplement: Supplementary file 1 [file pone.0359280.s001.pdf]

Referee Report for

Title: BORNOGICAL STABILIZATION FAMILIES AND OSCILLATION DYNAMICS OF CLOSED-SET SEQUENCES

Manuscript ID: PONE-D-26-20806

Submitted to PLOS ONE

The manuscript deals with the asymptotic behaviour of sequences of closed sets in metric spaces. The authors introduce a sequence-generated bornological structure. More precisely, they do not start with a fixed bornology. They start with a sequence of closed sets and then determine the subsets on which the sequence has vanishing internal oscillation.

The paper is built on three basic notions. The first one is the localized excess functional

$$e_S(A, C) = \sup_{a \in A \cap S} d(a, C),$$

with the usual convention for the empty trace. The second one is the symmetric localized oscillation gauge

$$\Delta_S(A, C) := \max\{e_S(A, C),\, e_S(C, A)\}.$$

The third one is the tail oscillation index

$$\omega_S(A_n) := \inf_{N \in \mathbb{N}} \sup_{m,n \geq N} \Delta_S(A_m, A_n).$$

Using these quantities, the authors define the stabilization family

$$\mathsf{S}_{\mathrm{stab}}(A_n) := \{S \subseteq X :\ \omega_S(A_n) = 0\}.$$

This construction is natural and useful. It gives a way to extract a bornological testing family from the sequence itself.

The main point of the paper is clear. In classical bornological convergence, one prescribes a bornology first and then studies convergence with respect to this family. In the present manuscript, the direction is reversed. The sequence itself generates the family of stabilizing regions. This makes the approach intrinsic. It is also different from Wijsman-type or pointwise distance-profile approaches, since the method is based on pairwise oscillation of traces and not on convergence of scalar distance functions.

The authors prove that the stabilization family is hereditary and stable under finite unions. Hence, when it covers the ambient metric space, it is a bornology. They also prove recovery theorems under robust stabilization and strong neighborhood trace-compactness. These results show how a closed local limit can be recovered on stabilizing regions. The compatibility theorem on a nested base and the global cluster-set theorem are also valuable parts of the paper. The maximality principle further clarifies the role of the generated bornology. It shows that any externally chosen bornology for which the sequence is already convergent must be contained in the generated stabilization family.

In my opinion, the manuscript satisfies the main scientific criteria of PLOS ONE. It presents original theoretical research. I did not see evidence that the main results have been published elsewhere. The paper is written in an intelligible mathematical style, and the results are supported by proofs and examples. Since the paper is theoretical, there are no experimental or statistical data to evaluate. The relevant standard is the logical correctness of the definitions, lemmas, theorems, and examples.

The examples are important for the manuscript and should be kept. They show that the proposed stabilization framework is not just a reformulation of known convergence notions. In particular, the examples illustrate stabilization without global Hausdorff convergence, the need for compactness in recovery, compatibility of local limits, and separation from pointwise distance-profile behaviour. These examples make the abstract theory much more transparent.

I recommend a minor revision before publication. The mathematical content is promising and essentially sound, but several points should be improved for precision and readability.

1. The authors should correct the numbering of definitions, lemmas, theorems, examples, and remarks. In the present version, many items appear as Definition 0.1, Theorem 0.1, Example 0.1, and so on. This gives the impression that the section counter has not been initialized correctly. The numbering should be consistent with the section structure of the paper.

2. There are several incomplete internal references. For example, the text contains expressions such as "Section" without a section number. These references should be completed. This is a minor editorial matter, but it is important for the final version.

3. Lemma 0.1 should be revised carefully. The intended triangle-type estimate is important for the later uniqueness and recovery arguments. However, in the localized setting one must be careful, because an approximate point of B chosen near a point of $A \cap S$ need not itself belong to S. Therefore, the estimate

$$e_S(A, C) \leq e_S(A, B) + e_S(B, C)$$

   should either be justified under an additional localization condition, or it should be replaced by a safer form. For example, the authors may use a global excess term in the second part, or a suitable thickened testing set. One possible way is to formulate the estimate with S^η, where the intermediate points are allowed to lie in a small neighbourhood of S. This is a local technical point, but it should be corrected since the lemma is used in the proof of uniqueness.

4. The authors should make the role of strong neighborhood trace-compactness more explicit. The paper correctly shows that vanishing oscillation alone does not guarantee recovery of a closed limit. This point is mathematically important. It would be useful to emphasize it once more in the conclusion.

5. The definition of strong neighborhood trace-compactness is central to the paper. I suggest adding one short explanatory sentence after the definition. The sentence may say that this condition prevents the local traces of the sequence from escaping to infinity while the oscillation on the testing region tends to zero.

6. In the statement of the global recovery theorem, the countable cofinal base condition should be explained briefly before the theorem. The theorem is correctly motivated by a diagonal argument, but the reader would benefit from one preparatory sentence explaining why a countable nested family of closed testing regions is imposed.

7. The conclusion is appropriate, but it can be strengthened. The authors may state explicitly that the paper provides a sequence-generated bornological method for closed-set dynamics and that this method is independent of both fixed-bornology convergence and pointwise distance-profile convergence.

8. The language of the manuscript is generally clear. Still, a final English check is recommended. Some sentences are long and can be divided into two shorter sentences. There is also a repeated sentence in one of the examples. These small changes will improve readability.

9. The data availability statement is acceptable for this type of work. Since the paper is a theoretical mathematical study and no datasets are generated or analysed, it is enough to state that all relevant mathematical material is contained in the manuscript.

10. After revising the manuscript, the authors must list the corrections with the title "Response text to the reviewer".

Overall, the manuscript develops a coherent and useful mathematical framework. The definitions are well motivated, the main results are logically connected, and the examples support the claims of the paper. The paper also fits the evaluation policy of *PLOS ONE*, because the main issue is scientific soundness rather than perceived impact. After the authors correct the localized estimate in Lemma 0.1 and make the minor editorial improvements listed above, I believe that the manuscript will be suitable for publication in *PLOS ONE*.

Therefore, I recommend **Minor Revision**.

In order to be sure the readability, I should review the revised form of the paper.
