# Supplementary material for: Bornological stabilization families and oscillation dynamics of closed-set sequences
Source: PLoS One. 2026 Sep 28;21(9):e0359280. doi: 10.1371/journal.pone.0359280 (PMC13618950; doi:10.1371/journal.pone.0359280)
Supplement: Supplementary file 3 [file pone.0359280.s002.pdf]

Response text to the reviewer

Manuscript: *Bornological stabilization families and oscillation dynamics of closed-set sequences*
Journal: PLOS ONE
Manuscript ID: PONE-D-26-20806R1

Professor Liangyun Chen
Academic Editor
PLOS ONE

Dear Professor Liangyun Chen,

We sincerely thank you and the reviewer for the careful reading of our manuscript and for the precise and constructive recommendations. We have revised the paper throughout. In particular, we have

(i) corrected the numbering of every theorem-like environment and completed all internal references;

(ii) replaced the invalid localized triangle estimate by a rigorously proved estimate on a metric enlargement of the test set and adjusted the subsequent argument that uses it;

(iii) clarified the indispensable role of strong neighborhood trace-compactness and the purpose of the increasing countable cofinal family;

(iv) strengthened the comparison with fixed-bornology and pointwise distance-function convergence; and

(v) completed a full language and consistency check, including removal of the repeated sentence noted in the report.

All changes are shown in the separate file entitled *Revised Manuscript with Track Changes*. Our point-by-point responses follow below.

Response text to the reviewer

R1.1. *The numbering of definitions, lemmas, theorems, examples, and remarks should be corrected and made consistent with the section structure.*

Addressed. All theorem-like environments are now numbered by section. For example, the corrected localized estimate is Lemma 3.1, the local recovery result is Theorem 3.1, the global recovery result is Theorem 3.3, and the examples are numbered 4.1–4.4. The former 0.x numbering no longer occurs.

Location in revised manuscript: Sections 2–4; all definitions, propositions, lemmas, theorems, corollaries, examples, and remarks.

R1.2. *Incomplete internal references, including occurrences of "Section" without a section number, should be completed.*

Addressed. We corrected every incomplete internal reference and checked all labels after compiling the manuscript. The organizational paragraph now refers explicitly to Sections 2, 3, and 4, and all references to propositions, lemmas, theorems, corollaries, and

examples resolve to their correct numbers. The final compilation contains no undefined citation or cross-reference.

Location in revised manuscript: Introduction; Sections 2–4; all internal cross-references throughout the manuscript.

R1.3. *Lemma 0.1 should be revised because the localized estimate* $e_S(A,C) \le e_S(A,B) + e_S(B,C)$ *is not valid without further localization: an intermediate point of* B *need not remain in* S*. A global term or a thickened test set should be used.*

Addressed. We agree with the reviewer and have removed the unsupported localized triangle inequality. It has been replaced by the following thickened-set estimate, now stated and proved as Lemma 3.1:

$$e_S(A,B) < r < \infty \quad \Longrightarrow \quad e_S(A,C) \le r + e_{S^r}(B,C).$$

The proof explicitly selects $y \in B$ with $d(x,y) < r$ for $x \in A \cap S$ and then observes that $y \in S^r$. The symmetric consequence is obtained by applying the same argument to both directions. We also rewrote Lemma 3.2 so that it invokes this corrected estimate on T^r; hence no later proof relies on the former invalid inequality.

Location in revised manuscript: Section 3, Lemma 3.1 and its proof; Lemma 3.2 and its proof.

R1.4. *The role of strong neighborhood trace-compactness should be made more explicit, and its necessity should be emphasized again in the conclusion.*

Addressed. The revised text now distinguishes clearly between the two roles of the hypotheses: neighborhood stabilization supplies localized tail Cauchy control, whereas strong neighborhood trace-compactness supplies the convergent subsequences needed to recover closed limits. We state explicitly after Theorem 3.3 and again in the conclusion that vanishing oscillation alone cannot prevent local traces from escaping every compact subset. Example 4.2 continues to show that recovery may fail when this compactness condition is absent.

Location in revised manuscript: Beginning of Section 3; paragraph following Theorem 3.3; Example 4.2; Conclusion and further directions.

R1.5. *A short explanatory sentence should be added after the definition of strong neighborhood trace-compactness to explain that it prevents local traces from escaping while oscillation tends to zero.*

Addressed. We added a remark immediately after the definition. It says that strong neighborhood trace-compactness prevents points of A_n lying near S from leaving every compact subset of X, and explains why using the same compact set for every neighborhood radius enables subsequential extraction.

Location in revised manuscript: Section 2, immediately after the definition of strong neighborhood trace-compactness.

R1.6. *The countable cofinal base condition should be explained briefly before the global recovery theorem.*

Addressed. We inserted preparatory text before the compatibility and global recovery results. It explains that countability coordinates the local comparisons, nesting makes the recovered sets compatible, and an increasing countable cofinal family provides a single ordered index for the diagonal construction. We also state that cofinality is what transfers convergence from the nested family to every stabilizing test set.

Location in revised manuscript: Section 3, the paragraphs immediately preceding Theorems 3.2 and 3.3.

R1.7. *The conclusion should state explicitly that the paper gives a sequence-generated bornological method independent of both fixed-bornology convergence and pointwise distance-profile convergence.*

Addressed. The conclusion now emphasizes that the testing bornology is derived from the sequence rather than prescribed in advance. It also states explicitly that the sequence-generated method is not a reformulation of either fixed-bornology convergence or pointwise distance-function convergence. The maximality result and the separation examples are summarized in the same section to support this distinction.

Location in revised manuscript: Conclusion and further directions; see also the Abstract and the final comparison paragraphs of the Introduction.

R1.8. *A final English check is recommended. Long sentences should be divided where appropriate, and the repeated sentence in one example should be removed.*

Addressed. We carefully edited the entire manuscript for grammar, sentence length, transitions, and terminological consistency. Repetitive and formulaic expressions were removed, and several long sentences were divided. The duplicated assertion concerning the failure of pointwise convergence was removed from Example 4.4. We also standardized the terminology used in the definitions, statements, proofs, abstract, introduction, and conclusion.

Location in revised manuscript: Entire manuscript; in particular, the Abstract, Introduction, Section 3, Example 4.4, and the Conclusion.

R1.9. *For this theoretical study, the data availability statement need only say that all relevant mathematical material is contained in the manuscript and that no datasets were generated or analyzed.*

Addressed. Following the journal's latest technical check, we removed the final sentence from the Data Availability Statement. The statement now reads: "This article reports no empirical or numerical data. No datasets were generated or analyzed during the current study. All definitions, assumptions, constructions, examples, and proofs needed to verify the results are contained in the manuscript." This change does not alter the mathematical content of the paper.

Location in revised manuscript: Data availability statement.

R1.10. *The corrections should be listed under the title "Response text to the reviewer."*

Addressed. The present point-by-point document bears the requested title *Response text to the reviewer.*

Location in revised manuscript: Title of this response document.

We thank you and the reviewer again for identifying the localized-estimate issue and for the recommendations that improved the exposition. We hope that the revised manuscript now addresses all concerns and is suitable for publication in *PLOS ONE*.

Sincerely,
Ömer Kişi
Corresponding author, on behalf of all authors
