# Supplementary material for: Bornological stabilization families and oscillation dynamics of closed-set sequences
Source: PLoS One. 2026 Sep 28;21(9):e0359280. doi: 10.1371/journal.pone.0359280 (PMC13618950; doi:10.1371/journal.pone.0359280)
Supplement: Peer review history

# Peer review: Decision Letter 0

**DOI:** 10.1371/journal.pone.0359280.r001

**Authors:** Liangyun Chen

28 Aug 2026

Dear Dr. Kişi,

Thank you for submitting your manuscript to PLOS One. After careful consideration, we feel that it has merit but does not fully meet PLOS One’s publication criteria as it currently stands. Therefore, we invite you to submit a revised version of the manuscript that addresses the points raised during the review process.

Please submit your revised manuscript by Oct 14 2026 11:59PM. If you will need more time than this to complete your revisions, please reply to this message or contact the journal office at plosone@plos.org. When you're ready to submit your revision, log on to https://www.editorialmanager.com/pone/ and select the 'Submissions Needing Revision' folder to locate your manuscript file.

- A letter that responds to each point raised by the academic editor and reviewer(s). You should upload this letter as a separate file labeled 'Response to Reviewers'.
- A marked-up copy of your manuscript that highlights changes made to the original version. You should upload this as a separate file labeled 'Revised Manuscript with Track Changes'.
- An unmarked version of your revised paper without tracked changes. You should upload this as a separate file labeled 'Manuscript'.

If you would like to make changes to your financial disclosure, please include your updated statement in your cover letter. Guidelines for resubmitting your figure files are available below the reviewer comments at the end of this letter.

If applicable, we recommend that you deposit your laboratory protocols in protocols.io to enhance the reproducibility of your results. Protocols.io assigns your protocol its own identifier (DOI) so that it can be cited independently in the future. For instructions see: https://journals.plos.org/plosone/s/submission-guidelines#loc-laboratory-protocols. Additionally, PLOS One offers an option for publishing peer-reviewed Lab Protocol articles, which describe protocols hosted on protocols.io. Read more information on sharing protocols at https://plos.org/protocols?utm_medium=editorial-email&utm_source=authorletters&utm_campaign=protocols.

As the corresponding author, your ORCID iD is verified in the submission system and will appear in the published article. PLOS supports the use of ORCID, and we encourage all coauthors to register for an ORCID iD and use it as well. Please encourage your coauthors to verify their ORCID iD within the submission system before final acceptance, as unverified ORCID iDs will not appear in the published article. *Only*  the individual author can complete the verification step; PLOS staff *cannot*  verify ORCID iDs on behalf of authors.

We look forward to receiving your revised manuscript.

Kind regards,

Liangyun Chen

Academic Editor

PLOS One

Journal Requirements:

When submitting your revision, we need you to address these additional requirements.

1.Please ensure that your manuscript meets PLOS ONE's style requirements, including those for file naming. The PLOS ONE style templates can be found at

https://journals.plos.org/plosone/s/file?id=wjVg/PLOSOne_formatting_sample_main_body.pdf and

https://journals.plos.org/plosone/s/file?id=ba62/PLOSOne_formatting_sample_title_authors_affiliations.pdf

2. We note that the grant information you provided in the ‘Funding Information’ and ‘Financial Disclosure’ sections do not match.

When you resubmit, please ensure that you provide the correct grant numbers for the awards you received for your study in the ‘Funding Information’ section.

3. Thank you for stating the following financial disclosure:

“Fujian Provincial Young and Middle-aged Teachers Education Research Project (Grant No.: JAT241239)”

Please state what role the funders took in the study.  If the funders had no role, please state: "The funders had no role in study design, data collection and analysis, decision to publish, or preparation of the manuscript."

If this statement is not correct you must amend it as needed.

Please include this amended Role of Funder statement in your cover letter; we will change the online submission form on your behalf.

4. Please provide a complete Data Availability Statement in the submission form, ensuring you include all necessary access information or a reason for why you are unable to make your data freely accessible. If your research concerns only data provided within your submission, please write "All data are in the manuscript and/or supporting information files" as your Data Availability Statement.

5. Please include captions for your Supporting Information files at the end of your manuscript, and update any in-text citations to match accordingly. Please see our Supporting Information guidelines for more information: http://journals.plos.org/plosone/s/supporting-information.

6. We are unable to open your Supporting Information file “PLOS ONE.zip”. Please kindly revise as necessary and re-upload

7. If the reviewer comments include a recommendation to cite specific previously published works, please review and evaluate these publications to determine whether they are relevant and should be cited. There is no requirement to cite these works unless the editor has indicated otherwise.

8. Please review your reference list to ensure that it is complete and correct. If you have cited papers that have been retracted, please include the rationale for doing so in the manuscript text, or remove these references and replace them with relevant current references. Any changes to the reference list should be mentioned in the rebuttal letter that accompanies your revised manuscript. If you need to cite a retracted article, indicate the article’s retracted status in the References list and also include a citation and full reference for the retraction notice.

[Note: HTML markup is below. Please do not edit.]

Reviewers' comments:

Reviewer's Responses to Questions

**Comments to the Author**

1. Is the manuscript technically sound, and do the data support the conclusions?

Reviewer #1: Yes

Reviewer #2: Yes

**********

2. Has the statistical analysis been performed appropriately and rigorously?

Reviewer #1: Yes

Reviewer #2: Yes

**********

3. Have the authors made all data underlying the findings in their manuscript fully available?

The PLOS Data policy

Reviewer #1: Yes

Reviewer #2: Yes

**********

4. Is the manuscript presented in an intelligible fashion and written in standard English?

Reviewer #1: Yes

Reviewer #2: Yes

**********

Reviewer #1: Comments on

"Bornological Stabilization Families and Oscillation Dynamics of Closed-Set Sequences"

Z.-P. Lin, M. Gurdal, and O. Kisi

Manuscript ID: PONE-D-26-20806

The manuscript submitted to PLOS ONE studies sequences of closed sets in metric spaces from a sequence-generated bornological point of view. Instead of fixing a bornology in advance, the authors introduce a stabilization family generated by the internal asymptotic oscillation of the sequence itself. This is formulated through a localized excess functional, a symmetric localized oscillation gauge, and a tail oscillation index. The paper then develops recovery results under robust stabilization and strong neighborhood trace-compactness, leading to local closed recovery, compatibility of local limits on nested testing regions, a global cluster set, and a maximality principle for the generated stabilization bornology.

In my opinion, the manuscript presents a coherent theoretical contribution. The topic is suitable for PLOS ONE, since the work should be evaluated primarily on scientific soundness, clarity, and technical correctness. The paper is mathematically motivated, the structure is logical, and the examples are useful in showing that the proposed stabilization framework is distinct from global Hausdorff convergence and from pointwise distance-profile approaches.

I recommend Minor Revision for the following reasons.

(1) Originality of the study. The manuscript presents original theoretical research. The main idea of extracting a bornological stabilization family directly from the asymptotic oscillation of a closed-set sequence appears to be a genuine contribution within the context of bornological and hyperspace-type convergence.

(2) Previously published results. Based on the manuscript and the cited literature, I did not find evidence that the reported results have been published elsewhere. The cited works are used to position the paper within bornological convergence, localized sequences, and related convergence structures.

(3) Technical standard of the analysis. This is a theoretical mathematical paper, and therefore the relevant standard is the correctness and completeness of the definitions, proofs, and examples. Most of the mathematical analysis is described in sufficient detail. However, Lemma 0.1 should be clarified carefully. The localized triangle-type estimate

e_{S}(A,C)≤e_{S}(A,B)+e_{S}(B,C)

is important for the later arguments. Since the proof uses approximation by points of B, the authors should make explicit why the localization on S is preserved, or alternatively state the estimate in a slightly modified localized or thickened form. This appears to be a technical clarification rather than a fundamental obstruction, but it should be addressed before publication.

(4) Support for the conclusions. The conclusions are appropriately supported by the mathematical results. The recovery theorems, compatibility result, maximality principle, and examples justify the main claims of the paper. The conclusion may be improved by explicitly emphasizing the role of strong neighborhood trace-compactness and by noting that vanishing oscillation alone is not sufficient for recovery.

(5) Clarity and English presentation. The manuscript is generally intelligible and written in standard mathematical English. The introduction clearly distinguishes the proposed sequence-generated viewpoint from classical fixed-bornology convergence and pointwise distance-profile approaches. Some minor editorial corrections are needed: several section references are incomplete, theorem and definition numbering appears as 0.1, 0.2, etc., and one sentence is repeated in Example 0.1.

(6) Ethics and research integrity. The manuscript is a theoretical mathematical study and does not involve human participants, animals, clinical material, field research, or experimental intervention. I did not identify any ethical concern.

(7) Data availability and reporting standards. The data availability statement is appropriate for a theoretical paper. Since no datasets were generated or analyzed, it is sufficient to state that all relevant mathematical arguments, proofs, and examples are contained in the manuscript.

The examples are useful and should be retained. Example 0.1 illustrates stabilization without global Hausdorff convergence; Example 0.2 shows the necessity of compactness-type assumptions; Example 0.3 clarifies compatibility of local limits on nested regions; and Example 0.4 effectively separates the proposed framework from pointwise distance-profile behavior.

In summary, the manuscript presents a sound and interesting theoretical framework. Subject to the clarification of Lemma 0.1 and the minor editorial corrections listed above, I believe that the manuscript will meet the publication criteria of PLOS ONE. Therefore, I recommend Minor Revision.

Reviewer #2: This is a well-written theoretical paper introducing a sequence-generated bornological framework for closed-set dynamics. The definitions are motivated, the examples are illuminating, and the results are logically sound. However, minor revisions are needed: correct the section numbering, fix incomplete internal references, revise Lemma 0.1 with a safer localization estimate, and clarify strong neighborhood trace-compactness. A final language polish is also recommended. No ethical or dual publication concerns. Recommend minor revision.

**********

what does this mean?). If published, this will include your full peer review and any attached files.

If you choose “no”, your identity will remain anonymous but your review may still be made public.

**Do you want your identity to be public for this peer review?**  For information about this choice, including consent withdrawal, please see our Privacy Policy

Reviewer #1: **Yes:**  Prof.Dr.Ayhan ESİ

Reviewer #2: No

**********

[NOTE: If reviewer comments were submitted as an attachment file, they will be attached to this email and accessible via the submission site. Please log into your account, locate the manuscript record, and check for the action link "View Attachments". If this link does not appear, there are no attachment files.]

To ensure your figures meet our technical requirements, please review our figure guidelines: https://journals.plos.org/plosone/s/figures

You may also use PLOS’s free figure tool, NAAS, to help you prepare publication quality figures: https://journals.plos.org/plosone/s/figures#loc-tools-for-figure-preparation.

NAAS will assess whether your figures meet our technical requirements by comparing each figure against our figure specifications.

Attachment Submitted filename: Review_PONE-D-26-20806.pdf

# Author comment: Author response to Decision Letter 1

**DOI:** 10.1371/journal.pone.0359280.r002

3 Sep 2026

Dear Professor Liangyun Chen,

We sincerely thank you and the reviewer for the careful evaluation of our manuscript and for the precise and constructive recommendations. A complete point-by-point response has been uploaded separately under “Response to Reviewers.” The principal revisions are summarized below.

R1.1. We corrected the numbering of all definitions, propositions, lemmas, theorems, corollaries, examples, and remarks so that it is consistent with the section structure.

R1.2. We completed and verified all internal cross-references. The revised manuscript contains no undefined citations or cross-references.

R1.3. We removed the invalid localized triangle inequality and replaced it with a rigorously proved thickened-set estimate. We also revised the subsequent argument so that no later proof relies on the former unsupported inequality.

R1.4. We clarified the indispensable role of strong neighborhood trace-compactness in the local and global recovery results and emphasized its necessity again in the conclusion.

R1.5. We added an explanatory remark immediately after the definition of strong neighborhood trace-compactness, explaining that this condition prevents local traces from escaping every compact subset while the oscillation tends to zero.

R1.6. We added preparatory text before the global recovery theorem to explain the roles of countability, nesting, and cofinality.

R1.7. We revised the conclusion to state explicitly that the sequence-generated bornological method is distinct from both fixed-bornology convergence and pointwise distance-function convergence.

R1.8. We completed a detailed English-language and consistency check, divided long sentences where appropriate, standardized the terminology, and removed the repeated sentence identified in the report.

R1.9. Following the journal’s technical check, we clarified the Data Availability Statement. It now confirms that this article reports no empirical or numerical data, that no datasets were generated or analyzed during the current study, and that all definitions, assumptions, constructions, examples, and proofs needed to verify the results are contained in the manuscript.

R1.10. The point-by-point response document bears the requested title “Response text to the reviewer.”

All modifications are highlighted in the separate file entitled “Revised Manuscript with Track Changes.” We thank you and the reviewer again for the recommendations that improved the mathematical precision and exposition of the manuscript.

Sincerely,

Ömer Kişi

Corresponding author, on behalf of all authors

Attachment Submitted filename: PONE_D_26_20806_Response_to_Reviewer_RV1__1_.pdf

# Peer review: Decision Letter 1

**DOI:** 10.1371/journal.pone.0359280.r003

**Authors:** Liangyun Chen

13 Sep 2026

Bornological stabilization families and oscillation dynamics of closed-set sequences

PONE-D-26-20806R1

Dear Dr. Kisi,

We’re pleased to inform you that your manuscript has been judged scientifically suitable for publication and will be formally accepted for publication once it meets all outstanding technical requirements.

Within one week, you’ll receive an e-mail detailing the required amendments. When these have been addressed, you’ll receive a formal acceptance letter and your manuscript will be scheduled for publication.

An invoice will be generated when your article is formally accepted. Please note, if your institution has a publishing partnership with PLOS and your article meets the relevant criteria, all or part of your publication costs will be covered. Please make sure your user information is up-to-date by logging into Editorial Manager at Editorial Manager® and clicking the ‘Update My Information' link at the top of the page. For questions related to billing, please contact billing support.

If your institution or institutions have a press office, please notify them about your upcoming paper to help maximize its impact. If they’ll be preparing press materials, please inform our press team as soon as possible -- no later than 48 hours after receiving the formal acceptance. Your manuscript will remain under strict press embargo until 2 pm Eastern Time on the date of publication. For more information, please contact onepress@plos.org.

Kind regards,

Liangyun Chen

Academic Editor

PLOS One

Additional Editor Comments (optional):

Reviewers' comments:

Reviewer's Responses to Questions

**Comments to the Author**

Reviewer #1: All comments have been addressed

Reviewer #2: All comments have been addressed

**********

2. Is the manuscript technically sound, and do the data support the conclusions?

Reviewer #1: Yes

Reviewer #2: Yes

**********

3. Has the statistical analysis been performed appropriately and rigorously?

Reviewer #1: Yes

Reviewer #2: Yes

**********

4. Have the authors made all data underlying the findings in their manuscript fully available?

The PLOS Data policy

Reviewer #1: Yes

Reviewer #2: Yes

**********

5. Is the manuscript presented in an intelligible fashion and written in standard English?

Reviewer #1: Yes

Reviewer #2: Yes

**********

Reviewer #1: I have read revised version again very carefully. Revised version of submitted paper is okay. Submitted paper could be accepted as it is.

Reviewer #2: The authors have adequately addressed all the comments raised in the previous round of review. The manuscript has been improved accordingly. I recommend acceptance of this manuscript.

**********

what does this mean?). If published, this will include your full peer review and any attached files.

If you choose “no”, your identity will remain anonymous but your review may still be made public.

**Do you want your identity to be public for this peer review?**  For information about this choice, including consent withdrawal, please see our Privacy Policy

Reviewer #1: **Yes:**  Prof.Dr.Ayhan ESİ

Reviewer #2: No

**********

# Editor report: Acceptance letter

**DOI:** 10.1371/journal.pone.0359280.r004

**Authors:** Liangyun Chen

PONE-D-26-20806R1

PLOS One

Dear Dr. Kişi,

I'm pleased to inform you that your manuscript has been deemed suitable for publication in PLOS One. Congratulations! Your manuscript is now being handed over to our production team.

At this stage, our production department will prepare your paper for publication. This includes ensuring the following:

* All references, tables, and figures are properly cited

* All relevant supporting information is included in the manuscript submission,

* There are no issues that prevent the paper from being properly typeset

You will receive further instructions from the production team, including instructions on how to review your proof when it is ready. Please keep in mind that we are working through a large volume of accepted articles, so please give us a few days to review your paper and let you know the next and final steps.

Lastly, if your institution or institutions have a press office, please let them know about your upcoming paper now to help maximize its impact. If they'll be preparing press materials, please inform our press team within the next 48 hours. Your manuscript will remain under strict press embargo until 2 pm Eastern Time on the date of publication. For more information, please contact onepress@plos.org.

You will receive an invoice from PLOS for your publication fee after your manuscript has reached the completed accept phase. If you receive an email requesting payment before acceptance or for any other service, this may be a phishing scheme. Learn how to identify phishing emails and protect your accounts at https://explore.plos.org/phishing.

If we can help with anything else, please email us at customercare@plos.org.

Thank you for submitting your work to PLOS One and supporting open access.

Kind regards,

PLOS One Editorial Office Staff

on behalf of

Dr. Liangyun Chen

Academic Editor

PLOS One
